# The association between regional adiposity, cognitive function, and dementia-related brain changes: a systematic review

**DOI:** 10.3389/fmed.2023.1160426

**Published:** 2023-06-30

**Authors:** Ethel Boccara, Sapir Golan, Michal Schnaider Beeri

**Affiliations:** ^1^Department of Psychology, Bar-Ilan University, Ramat Gan, Israel; ^2^The Joseph Sagol Neuroscience Center, Sheba Medical Center, Tel HaShomer, Israel; ^3^Sackler Faculty of Medicine, Tel Aviv University, Tel Aviv, Israel; ^4^Department of Psychiatry, Icahn School of Medicine at Mount Sinai, New York, NY, United States

**Keywords:** regional adiposity, fat distribution, visceral, liver, cognition, brain, dementia, Alzheimer’s disease

## Abstract

**Background:**

Adiposity has been previously associated with cognitive impairment and Alzheimer’s disease and related disorders (ADRD). Body mass index (BMI) is the most common measure of global adiposity, but inconsistent results were found since it is a global measurement. BMI does not represent regional fat distribution which differs between sexes, race, and age. Regional fat distribution may contribute differently to cognitive decline and Alzheimer’s disease (AD)-related brain changes. Fat-specific targeted therapies could lead to personalized improvement of cognition. The goal of this systematic review is to explore whether regional fat depots, rather than central obesity, should be used to understand the mechanism underlying the association between adiposity and brain.

**Methods:**

This systematic review included 33 studies in the English language, conducted in humans aged 18 years and over with assessment of regional adiposity, cognitive function, dementia, and brain measures. We included only studies that have assessed regional adiposity using imaging technics and excluded studies that were review articles, abstract only or letters to editor. Studies on children and adolescents, animal studies, and studies of patients with gastrointestinal diseases were excluded. PubMed, PsychInfo and web of science were used as electronic databases for literature search until November 2022.

**Results:**

Based on the currently available literature, the findings suggest that different regional fat depots are likely associated with increased risk of cognitive impairment, brain changes and dementia, especially AD. However, different regional fat depots can have different cognitive outcomes and affect the brain differently. Visceral adipose tissue (VAT) was the most studied regional fat, along with liver fat through non-alcoholic fatty liver disease (NAFLD). Pancreatic fat was the least studied regional fat.

**Conclusion:**

Regional adiposity, which is modifiable, may explain discrepancies in associations of global adiposity, brain, and cognition. Specific regional fat depots lead to abnormal secretion of adipose factors which in turn may penetrate the blood brain barrier leading to brain damage and to cognitive decline.

## Background

Adiposity refers to the state of being excessively overweight or obese, which is typically caused by an excessive accumulation of body fat and is strongly associated with type II diabetes (T2D), cardiovascular disease, hypertension, and hyperlipidemia (1). Adiposity has been previously associated with cognitive impairment and Alzheimer’s disease and related disorders (ADRD) (2, 3). Characterization of how adiposity impacts ADRD is necessary because adiposity prevention and treatment could be a safe, efficacious approach to prevent ADRD. Body mass index (BMI) is the most common measure of global adiposity. BMI is calculated as weight (kg) divided by the square of height (*m*^2^). Obesity is defined as BMI ≥30 kg/m2. Higher BMI in midlife has been associated with poor cognitive outcomes in late life (4, 5). Poorer performance in executive function (6) as well as working memory (7) and verbal fluency (8) have been consistently associated with higher BMI. We have shown that greater weight variability in midlife is associated with an increased risk of dementia three decades later (4). We have also found evidence for associations of greater variability in BMI over time with faster cognitive decline in late life (9). However, inconsistent results were found in old-age where high BMI has been associated with both higher risk (10, 11) but also lower risk (5, 12–14) for dementia. This non-linear association is often attributed to the fact that weight-loss can precede the development of AD (15–17) along with sarcopenia, the loss of skeletal muscle mass and function (18–21).

One explanation for these discrepancies is that BMI may not be a good measure of adiposity (22) since it represents global, rather than regional fat distribution (23, 24) especially in old age (25, 26). While BMI can assess excessive body fat, it does not account for different regional fat depots and muscle mass. Regional fat depots can be at the origin of different metabolic risks (27) since different fat depots have specific metabolic and hormonal characteristics. Previous data suggest that some obese individuals are metabolically healthy, free from high cardiovascular disease and with a normal metabolic risk profile. Contrariwise, metabolically unhealthy individuals with normal weight can be at high risk of cardiovascular disease (27, 28). Investigation of regional fat in these unique populations may shed light into these discrepancies. For them, little is known about the association of regional fat, brain, and cognition and such research is warranted.

Previous studies have shown that visceral adipose tissue (VAT) rather than obesity calculated by BMI, was associated with cardiovascular disease risk and metabolic syndrome (29, 30) while lower amounts of lower-body fat mass (gluteo-femoral) were also found to be a determinant of cardiometabolic diseases (27, 31). Ectopic fat, which refers to the accumulation of fat in areas where it is not normally found, such as the liver, and pancreas, is strongly associated with obesity and insulin resistance (32, 33). However, previous research has shown that nonalcoholic fatty liver disease (NAFLD) was associated with the metabolic syndrome regardless of central obesity assessed by BMI and insulin resistance (34). These findings suggest that some adipose tissues are deleterious while others have a protective role, indicating that although BMI is a widely used tool to assess central obesity, different regional fat depots may have different roles in cardiovascular risk factors and disease, themselves associated with ADRD (35, 36).

Furthermore, the appropriateness of BMI as a phenotypic marker of adiposity across populations differing in race and ethnicity is now questioned (37). Additionally, BMI does not account for sex differences in excessive fat. Women tend to have more fat than man, but the fat distribution is different between the sexes (38). This leaves critical gaps in knowledge about specific adiposity phenotypes that may differentially affect ADRD risk and neuropathology in old age. Body fat distribution has been linked to cognitive function in old age. Specific anatomical location of stored excess fat, including VAT, subcutaneous adipose tissue (SAT), or fat stored within the organs has been linked to cognition (39–41). Greater VAT was associated with lower delayed memory and language scores suggesting that regional adiposity may be linked to specific cognitive domains (39). There is evidence that the development of cognitive decline seems more strongly related to specific body fat distribution than to BMI (40). Exploring regional adiposity might contribute to the understanding of the mechanisms underlying the relationship of adiposity, cognitive function, and associated brain changes. Indeed, increased fat mass in different abdominal regions contributes to the dysregulation of adipokine secretion, increase of inflammation and release of fatty acids into the circulation (42), different human fat pools could lead to different interventions. Therefore, it is important to use additional measurements and assessments to obtain a more accurate picture of an individual’s overall health and body composition.

Thus, the aim of this review is to explore the association between regional adiposity, different fat depots, cognitive function, and associated brain changes.

## Method

### Eligibility criteria

The present systematic review included studies of all designs if they were in the English language, conducted in humans aged 18 years and over with assessment of regional adiposity, cognitive function, dementia, and brain changes.

We included studies that have assessed regional adiposity using imaging techniques. There are several methods for measuring regional adipose tissues (43). Bioelectrical impedance analysis has been widely used to assess different fat tissues including VAT (44) but was shown to be less accurate in differentiating between the abdominal fat tissues compared to imaging techniques such as computed tomography (CT) (45), magnetic resonance imaging (MRI) (46) and dual energy x-ray absorptiometry (DEXA) (43, 47). Abdominal Ultrasound (US) is an accurate imaging technique for the detection of fatty liver (48). Therefore, we kept studies that used only imaging techniques to assess regional fat depots. Those include abdominal CT, DEXA, abdominal MRI, and abdominal US.

Studies were excluded if they were review articles, abstract only or letters to editor. Studies on children and adolescents and animal studies were excluded. Studies on patients with severe gastrointestinal diseases were excluded because significant inflammatory changes in the intestine can affect body composition (49, 50).

### Search strategy

The review followed the Preferred Reporting Items for Systematic Reviews and Meta-Analyses (PRISMA) guidelines (51). PubMed, PsychInfo, and web of science were used as electronic databases for literature search until November 2022. The search terms were: (“Cognitive decline” OR “cognition” OR “memory” OR “executive function” OR “cognitive” OR “Alzheimer’s disease” OR “Dementia” AND “regional adiposity”) further research was done replacing the term “regional adiposity” with “visceral adiposity,” “subcutaneous adipose tissue,” “hepatic fat,” “fatty liver,” “pancreatic fat,” “pancreatic steatosis,” “fat distribution.”

### Study selection

**Figure 1:**
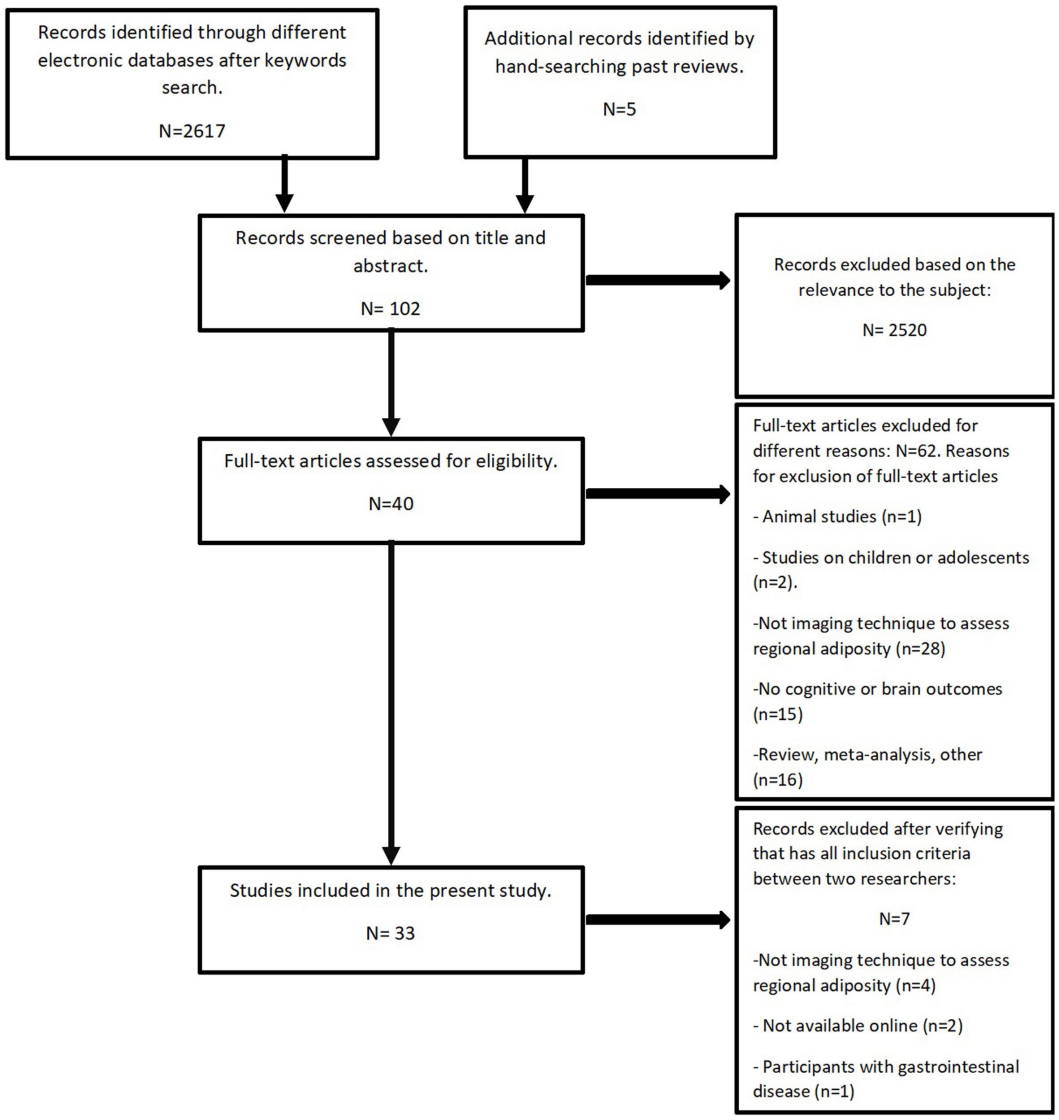
Flow chart.

Title and abstract screening were carried out by one researcher (EB), duplicates and articles which did not meet the eligibility criteria were excluded. Articles which did not investigate regional adiposity and cognition or brain changes, were conducted on children or which used adiposity assessment that did not include imaging techniques were excluded. Full text screening was conducted independently by two researchers. Articles that fulfilled the selection criteria after the full text was read, were included in this systematic review. The study selection is shown in the flowchart in Figure 1.

The search strategy resulted in 2621 articles. From these, 2,520 articles were deemed ineligible after title and abstract screening. A hundred and one studies were eligible for full-text screening and 69 were excluded for the following reasons: measure of adiposity not with imaging technique (*n* = 32), meta-analysis or review (*n* = 16), no cognitive or brain outcomes (*n* = 15), not available online (*n* = 2), participants with inflammatory bowel disease (*n* = 1), animal study (*n* = 1), and studies on children or adolescents (*n* = 2). One study was found after independent research. Hence, 33 observational studies were included in this systematic review.

### Data extraction and grouping

Data was extracted from 40 studies by one researcher and then re-checked by a second researcher. Both researchers agreed on including 33 studies in the present study based on inclusion criteria previously stated. Data extraction included the following: author, year published, country of the study, population characteristics including number of participants, number of women, mean age, which regions of adiposity were assessed, the technique of assessment, the measurement methods for cognitive function or brain measures. These findings are shown in Tables 1, 2.

**Table 1:** Characteristics of the included studies about regional adiposity and cognition.

Ref.	Article	Origin of population	Nb of participants	Women %	Age Mean years (SD)	Regional Adiposity	Method of acquisition	Measures of Cognition	Cognitive tests	Brain MRI	Brain parts
(52)	Anand et al. (2022)	Canada Poland	*N* = 6,773	56.4%	57.8 (8.8)	VAT	Abdominal MRI	Global cognitive function	DSST MoCA	Brain MRI	WMH BI
(53)	Zsido et al. (2019)	Germany	*N* = 974	48.6%	50.7 (15.6)	VAT	Abdominal MRI	Memory performance	CERAD	MPRAGE	Brain network covariance
(54)	Isaac et al. (2011)	Singapore	*N* = 184	52.2%	67.9 (6.4)	VAT SAT	Abdominal MRI	Six cognitive domains: attention, verbal memory, visuo-spatial memory, executive functioning, speed of processing, language	MMSE DSST Spatial span task RAVLT WMS-III Verbal fluency test Design fluency test, TMT A TMT B SDMT Object and action naming battery	Brain MRI	Total brain volume, Hippocampal volume, Ventricular volume, and Cortical thickness.
(55)	Kim et al. (2022)	Korea	*N* = 54	63%	66.4 (8.4)	VAT metabolism	PET-CT FDG	Global cognitive function	MMSE	PET-CT Brain MRI	Amyloid burden by PET-CT WMH
(56)	Kanaya et al. (2009)	USA	N = 3,054	51.2%	73.6 (2.8)	VAT SAT	Abdominal CT	Global cognitive function	3MS		
(57)	Yoon et al. (2012)	Korea	*N* = 250	54%	66.4 (2.8)	VAT SAT	Abdominal CT	Global cognitive function	MMSE		
(58)	Kim et al. (2019)	Korea	*N* = 110	41%	63.0 (6.4)	VAT	Abdominal CT	Global cognitive function	MMSE	Brain FDG PET/CT scan	PALZ score
(59)	Hsu et al. (2016)	USA	*N* = 604	59.9%	57.7 (9.3)	VAT SAT PAT	Abdominal CT	Global cognitive function Executive function	3MS DSST RAVLT Stroop Task COWA	Brain MRI	WMV GMV WM lesions hippocampal GMV, hippocampal WMV,
(60)	Gerber et al. (2021)	USA	*N* = 2,809	57%	50.1 (3.6)	VAT SAT LA	Abdominal CT	Attention, working memory, psychomotor speed, executive function, Verbal memory.	DSST RAVLT Stroop Task		
(21)	Spauwen et al. (2017)	Iceland	*N* = 5,169	57.1%	76.4(5.5)	VAT SAT Thigh SAT	Abdominal and thigh CT	Dementia	DSM-IV		
(61)	Meng et al. (2020)	USA	*N* = 30	50%	69.9 (7.1)	VAT	DEXA	Global cognitive function N-back cognitive function	MoCA		
(62)	Pasha et al. (2017)	USA	*N* = 126	54.8%	49.1 (6.6)	VAT	DEXA	Global cognitive function Memory Executive function	MMSE WASI-II FSIQ CVLT-II TMT A TMT B WASI-III DSST Stroop task	Brain MRI	WM lesion
(63)	Cannavale et al. (2021)	USA	*N* = 115	66.1%	33.8 (6.1)	VAT	DEXA	Attentional inhibitory control	Eriksen Flanker task		
(64)	Mazzoccoli et al. (2014)	Italy	*N* = 71	50.7%	72.7 (7.1)	EAT	Transthoracic echocardiography	Global cognitive function	MMSE		
(65)	Verrusio et al. (2019)	Italy	*N* = 65	52.3%	72.1 (8.9)	EAT	Transthoracic echocardiography	Global cognitive function	MMSE		
(66)	Wang et al. (2022)	China	*N* = 5,129	61.79%	71.06 (5.97)	NAFLD	Transabdominal US	Dementia	DSM IV		
(67)	Filipovic et al. (2018)	Serbia	*N* = 76	42.1%	47.5 (6.5)	Hepatic fat	Abdominal US	Global cognitive function	MoCA	Brain MRI	TCBV, lateral ventricle volume, GMV WMV
(68)	Liu et al. (2022)	China	*N* = 1,651	51.2%	53.4 (8.4)	NAFLD	Abdominal US	Global cognitive function	MMSE		
(69)	Yu et al. (2022)	USA	*N* = 4,973	50.3%	37.2 (11.1)	NAFLD	Abdominal US	Global cognitive function	SDLT, SRTT, DSST		
(70)	Celikbilek et al. (2018)	Turkey	*N* = 143	62.9%	45.3 (9.7)	NAFLD	Abdominal US	Global cognitive function	MoCA		
(71)	Seo et al. (2016)	USA	*N* = 4,472	52.5%	37.3(0.3)	NAFLD	Abdominal US	Global cognitive function	SRTT, DSST, SDLT		
(72)	Moretti et al. (2022)	Italy	*N* = 285	48.1%	76 (1)	NAFLD	Abdominal US	Frontal dementia Anxiety Apathy Global behavioral symptoms Quality of life in dementia scale	FAB, HAM-A, AES-C, NPI,QUALID		

VAT, visceral adipose tissue; SAT, subcutaneous adipose tissue; PAT, pericardial adipose tissue; NAFLD, non-alcoholic fatty liver disease; LA, liver attenuation; EAT, epicardial adipose tissue; MRI, magnetic resonance imaging; MPRAGE, magnetization prepared – rapid gradient echo; DEXA, dual- energy X- ray absorptiometry; CT, computed tomography; PET-CT FDG, positron emission tomography – CT F-fluorodeoxyglucose; US, ultrasound; Fmri, functional MRI; WMH, white matter hyperintensities; BI, brain infarctions; WMV, white matter volume; GMV, gray matter volume; CSF, cerebrospinal fluid; FA, fractional anisotropy; LI, lacunar infarct; TCBV, total cranial brain volume; WMHV, WMH volume; GM, gray matter; WM, white matter; CBF, cerebral blood flow; MBs, microbleeds; DSST, digit symbol substitution test; MoCA, the Montreal cognitive assessment; MMSE- the mini-mental state examination; 3MS, the modified mini-mental state test; SDMT, the symbol digit modalities test; RAVLT, the Rey auditory verbal learning test; COWA, controlled oral word association test; CERAD, the consortium to establish a registry for Alzheimer’s disease; FAB, frontal assessment battery; BDI, beck depression inventory test; HAM-A, Hamilton anxiety rating scale; AES-C, the apathy evaluation scale; SDLT, serial digit learning test; SRTT, the simple reaction time task; NPI, the neuropsychiatric inventory questionnaire; QUALID, quality of life in late stage dementia; DSM IV, diagnostic and statistical manual of mental disorders IV.

**Table 2:** Characteristics of the included studies about regional adiposity and brain.

Ref	Article	Origin of population	Nb of participants	Women %	Age Mean years (SD)	Regional adiposity	Method of acquisition	Measures of cognition	Cognitive tests	Brain MRI	Brain regions
(53)	Zsido et al. (2019)	Germany	*N* = 974	48.6%	50.68 (15.6)	VAT	Abdominal MRI	Memory performance	CERAD	MPRAGE MRI	Brain network covariance
(41)	Beller et al. (2019)	Germany	*N* = 351	43.3%	56.2 (± 9.0)	VAT SAT Hepatic fat pancreatic fat	Abdominal MRI			Brain MRI	Cingulate gyri, hippocampus, temporal lobe
52	Anand et al. (2022)	Canada Poland	*N* = 6,773	56.4%	57.8 (8.8)	VAT	Abdominal MRI	Global cognitive function	DSST MoCA	Brain MRI	WMH BI
(54)	Isaac et al. (2011)	Singapore	*N* = 184	52.2%	67.9 (6.4)	VAT SAT	Abdominal MRI	Six cognitive domains: attention, verbal memory, visuo-spatial memory, executive functioning, speed of processing, and language was used.	MMSE, digit span, spatial span task (echsler), RAVLT, WMS-III, verbal fluency test, design fluency test, trail making test B and A, SDMT, object and action naming battery	Brain MRI	Total brain volume, hippocampal volume, ventricular volume, and cortical thickness.
(73)	Raschpichler et al. (2016)	Germany	*N* = 100	42%	51.7 (16.4)	VAT SAT	Abdominal MRI			Brain MRI Fmri	Gray matter density
(74)	Cardenas et al. (2020)	Spain	*N* = 23	0%	36.79 (8)	VAT	DEXA			Brain MRI	WM FA
(62)	Pasha et al. (2017)	USA	*N* = 126	54.8%	49.1 (6.6)	VAT	DEXA	Global cognitive function Memory Executive function	MMSE WASI-II FSIQ subtest, California Verbal Learning Test-II (CVLT-II), Trail Making Tests A and B, Wechsler Adult Intelligence Scale III (WASI-III) Digit Span subtest -Stroop color-word subtest.	Brain MRI	WM lesion
(75)	Kaur et al. (2015)	USA	*N* = 103	52.4%	49.63 (6.47)	VAT	DEXA			Brain MRI	Cortical thickness
(55)	Kim et al. (2022)	Korea	*N* = 54	63%	66.4 (8.4)	VAT	PET-CT FDG	Global cognitive function	MMSE	PET-CT Brain MRI	Amyloid burden by PET-CT WMH
(59)	Hsu et al. (2016)	USA	*N* = 604	59.9%	57.7(9.3)	VAT SAT PAT	Abdominal CT	Global cognitive function Executive function	3MS Digit Symbol Coding, the RAVLT-delayed recall, the Stroop Task COWA	Brain MRI	WMV GMV WM lesions hippocampal GMV, hippocampal WMV,
(58)	Kim et al. (2019)	Korea	*N* = 110	41%	63.0 (6.4)	VAT	Abdominal CT	Global cognitive function	MMSE	Brain FDG PET/CT scan	PALZ score
(76)	Lee et. al. (2021)	Korea	*N* = 1,209	52.5%	63.6 (6.9)	VAT	Abdominal CT			Brain MRI	CSF,frontal, parietal,temporal,occipital, subcortical and cerebellum
(77)	Kim et al. (2017)	Korea	*N* = 1991	37.4%	50.3 (5.2)	VAT SAT	Abdominal CT			Brain MRI	WMH LI
(78)	Debette et al. (2010)	USA	*N* = 733	53%	64 (9)	VAT SAT	Abdominal CT			Brain MRI	TCBV, temporal horn volume, WMHV BI
(79)	VanWagner et al. (2017)	USA	*N* = 505	55.8%	50.1(3.6)	VAT SAT LA	Abdominal CT			Brain MRI pCASL	Total brain tissue volume GM WM CSF CBF
(80)	Weinstein et al. (2018)	USA	*N* = 766	53.5%	67 (9)	Hepatic fat	Abdominal CT			Brain MRI	TCBV, hippocampal volume WMHV BI
(81)	Weinstein et al. (2022)	USA	*N* = 169;	43%	52 (9)	NAFLD	Abdominal CT			PET-CT	Regional Amyloid-β and Tau Pathology
(67)	Filipovic et al. (2018)	Serbia	*N* = 76	42.1%	47.5 (6.5)	Hepatic fat	Abdominal US	Global cognitive function	MoCA	Brain MRI	TCBV, lateral ventricle volume, GMV WMV
(82)	Jang et al. (2019)	Korea	*N* = 1,260	49.2%	63.7(6.8)	NAFLD	Abdominal US	Global cognitive function	MMSE	Brain MRI	CSVD WMH Lacunes Microbleeds (MBs)

VAT, visceral adipose tissue; SAT, subcutaneous adipose tissue; PAT, pericardial adipose tissue; NAFLD, non-alcoholic fatty liver disease; LA, liver attenuation; EAT, epicardial adipose tissue; MRI, magnetic resonance imaging; MPRAGE, magnetization prepared – rapid gradient echo; DEXA, dual- energy X- ray absorptiometry; CT, computed tomography; PET-CT FDG, positron emission tomography – CT F-fluorodeoxyglucose; US, ultrasound; Fmri, functional MRI; Pcasl, pseudo-continuous arterial spin labeling; WMH, white matter hyperintensities; BI, brain infarctions; WMV, white matter volume; GMV, gray matter volume; CSF, cerebrospinal fluid; FA, fractional anisotropy; LI, lacunar infarct; TCBV, total cranial brain volume; WMHV, WMH volume; GM, gray matter; WM, white matter; CBF, cerebral blood flow; MBs, microbleeds; DSST, digit symbol substitution test; MoCA, the Montreal cognitive assessment; MMSE, the mini-mental state examination; 3MS, the modified mini-mental state test; SDMT, the symbol digit modalities test; RAVLT, the Rey auditory verbal learning test; COWA, Controlled Oral Word Association Test; CERAD, the consortium to establish a registry for Alzheimer’s disease; FAB, frontal assessment battery; BDI, beck depression inventory test; HAM-A, Hamilton anxiety rating scale; AES-C, the apathy evaluation scale; SDLT, serial digit learning test; SRTT, the simple reaction time task; NPI, the neuropsychiatric inventory questionnaire; QUALID, quality of life in late stage dementia; DSM IV- diagnostic and statistical manual of mental disorders IV.

### Study characteristics

The data extracted from the 33 studies included in this review (36, 41, 52–82) are presented in Tables 1, 2. All studies had an observational design and were cross-sectional studies except for three longitudinal cognition studies (56, 60, 68). The articles were published between 2009 and 2022. The studies were conducted in the United States, Korea, Canada, Poland, Germany, Singapore, Serbia, Sweden, Turkey, Italy, Iceland and Spain, and included a total of 44,327 participants aged between 18 and 89 years (mean 54.11 years).

### Statistical analysis

Results including the association of different regional fat depots with cognition and brain changes are presented narratively. For qualitative analysis, differences in measures between higher regional fat depots and control groups or the appropriate results were reported for individual studies. Data is considered statistically significant if the results reported have a value of *p* smaller than 0.05.

## Results

### Visceral adiposity and cognition

According to our search, we have grouped 13 studies assessing VAT and cognitive outcomes, nine (52, 54–59, 61, 62) of them using global cognition measurement such as Mini-mental state examination (MMSE) (83), modified MMSE (3MS) (84) and the Montreal Cognitive Assessment, (MoCA) (85). All studies were cross-sectional (52–55, 57–63) except for two longitudinal (36, 56). Among them, six studies have also assessed SAT and cognitive outcomes (36, 54, 56, 57, 59, 60). While in some studies, absence of significant associations of VAT (36, 55, 56, 58, 59, 62) and SAT (57, 59) with cognitive functioning have been shown, in most of our search, higher VAT was found to be associated with lower cognitive scores (52–54, 57, 60, 61, 63). In studies including both VAT and SAT measures, variable results were found. SAT and VAT were associated with lower verbal memory; VAT was independently associated with lower cognition when accounting for SAT but not the other way around (54). In another study, higher VAT but not higher SAT was associated with poor cognitive functioning (57). Interestingly, sex had opposite effects in these associations. Higher SAT (but not VAT) was associated with worsening cognitive functioning after 7 years in men (56). In contrast, in women higher levels of SAT and VAT were associated with less cognitive decline over the years (56). In another study, higher SAT and subcutaneous thigh fat were associated with a decreased likelihood of dementia in women (36). The impact of regional fat depots on cognitive functions can be found in Table 3.

**Table 3:** Cognition and regional adiposity in studies included in the systematic review: the table is ordered by fat depots from the newest to the oldest publication.

Regional adiposity	Cognitive variables	Results	References
Visceral adipose tissue (VAT)	DSST MoCA	Higher VAT was associated with significantly lower DSST scores but not with MoCA scores.	Anand et al. (52)
	Attentional inhibitory control [Eriksen Flanker task]	High VAT was associated with poorer selective attention.	Cannavale et al. (63)
	DSST RAVLT STROOP TASK	VAT was associated with lower cognitive function at baseline and follow-up.	Gerber et al. (60)
	MoCA N-back (Reaction time and accuracy.)	Higher VAT associated with higher MoCA score. Higher VAT is significantly associated with longer reaction time. No association found between VAT and response accuracy.	Meng et al. (61)
	Memory network covariance	Higher VAT was associated with lower memory network covariance in men. This association was not found in women.	Zsido et al. (53)
	Dementia	No significant association of VAT with dementia.	Spauwen et al. (36)
	MMSE Memory Executive function [WASI-II FSIQ CVLT-II TMT-A TMT-B DIGIT SPAN subtest STROOP TASK]	No significant associations were found between VAT and cognitive function in any of the tests or the cognitive domains.	Pasha et al. (62)
	3MS DSST RAVLT STROOP TASK COWA	No significant associations were found between VAT and any of the cognitive tests.	Hsu et al. (59)
	MMSE	Higher VAT was associated with poorer cognitive performance among subjects younger than 70 years old. No significant associations were found for older subject.	Yoon et al. (57)
	Attention Verbal Memory Visuo-spatial Memory Executive functioning Speed of processing Language	Higher VAT was significantly associated with lower attention and verbal memory scores. There were no significant associations with the other cognitive domains.	Isaac et al. (54)
	Modified MMSE (3MS)	Higher level of VAT was significantly associated with worse cognitive function in men but not in women.	Kanaya et al. (56)
VAT metabolism	MMSE K-BNT score	Higher VAT metabolism was significantly associated with lower K-BNT score. No significant associations were found with MMSE.	Kim et al. (55)
Subcutaneous adipose tissue (SAT)	DSST RAVLT STROOP TASK	SAT was associated with lower cognitive function at baseline and follow-up	Gerber et al. (60)
	Dementia	Higher amounts of SAT and subcutaneous thigh fat were associated with a decreased likelihood of dementia in women.	Spauwen et al. (41)
	3MS DSST RAVLT STROOP TASK COWA	SAT was not associated with any of the cognitive tests.	Hsu et al. (59)
	MMSE	SAT was not related to poorer cognitive performance among younger and older adults.	Yoon et al. (57)
	Attention Verbal Memory Visuo-spatial Memory Executive functioning Speed of processing Language	Higher SAT was significantly associated with lower attention and verbal memory scores.	Isaac et al. (54)
	Modified MMSE (3MS)	Higher level of SAT was significantly associated with worse cognitive function in men but not in women.	Kanaya et al. (56)
Hepatic fat	MMSE	Older adults with NAFLD had higher 4-year incidence of cognitive impairment than those without NAFLD.	Liu et al. (68)
	SDLT SRTT DSST	NAFLD was significantly associated with high risk of low SDLT, SRTT and DSST scores. The fully adjusted model remained significant only for SRTT.	Yu et al. (69)
	FAB HAM-A NPI QUALID	NAFLD was strongly associated with vascular dementia.	Moretti et al. (72)
	Dementia	Moderate to severe NAFLD was associated with increased likelihood of all cause dementia.	Wang et al. (66)
	DSST RAVLT STROOP TASK	NAFLD was associated with lower cognitive function	Gerber et al. (60)
	MoCA	NAFLD patients had lower MoCA scores.	Filipovic et al. (67)
	MoCA	MoCA scores were significantly lower in participants with NAFLD than in healthy controls	Celikbilek et al. (70)
	SDLT SRTT DSST	Compared to healthy participants, NAFLD was significantly associated with high risk of low SDLT, SRTT and DSST scores. In a fully adjusted model only the associations with SDTL remained significant.	Seo et al. (71)
Pericardial adipose tissue (PAT)	3MS DSST RAVLT STROOP TASK COWA	No significant association were found between pericardial adipose tissue and cognitive functions.	Hsu et al. (59)
Epicardial adipose tissue (EAT)	MMSE	High epicardial adipose tissue thickness was associated with lower cognitive function.	Verrusio et al. (65)
	MMSE	High epicardial adipose tissue thickness was associated with lower cognitive function.	Mazzoccoli et al. (64)

VAT, visceral adipose tissue; SAT, subcutaneous adipose tissue; PAT, pericardial adipose tissue; NAFLD, non-alcoholic fatty liver disease; EAT, epicardial adipose tissue; DSST, digit symbol substitution test; MoCA, the Montreal cognitive assessment; MMSE, the mini-mental state examination; 3MS, the modified mini-mental state test; SDMT, the symbol digit modalities test; RAVLT, the Rey auditory verbal learning test; COWA, controlled oral word association test; CERAD, the consortium to establish a registry for Alzheimer’s disease; FAB, frontal assessment battery; BDI, beck depression inventory test; HAM-A, Hamilton Anxiety rating scale; AES-C, the apathy evaluation scale; SDLT, serial digit learning Test; SRTT, the simple reaction time task; NPI, the neuropsychiatric inventory questionnaire; QUALID, quality of life in late stage dementia; WASI-II, Wechsler abbreviated intelligence scale; FSIQ, full scale intelligence quotient; CVLT-II, California verbal learning test; TMT, trail making test.

### Visceral adiposity and brain changes

Twelve articles reported on the association between VAT and SAT and different brain changes (41, 52–54, 59, 62, 73–78). The association between different brain compartments and regional fat depots can be found in Table 4. All the papers have used structural brain measures via brain MRI (41, 52–54, 59, 62, 73–78), while only one study used also functional measures via functional MRI (fMRI) to assess degree of connectivity (eigenvector centrality, EC) (73). This study showed that high VAT was associated with lower cerebellar structure (gray matter density) as well as lower degree of connectivity of the cerebellum with other brain regions in younger subjects; no associations were found in older individuals (73). These results suggest that the relationship of increased VAT with reduced gray matter density and reduced connectivity in the cerebellum, which is involved in cognitive function, are age-dependent (73). Structural measures have shown that higher VAT and SAT were associated with smaller total brain volume (78). Moreover, elevated VAT was correlated with cortical thinning especially with lower hippocampal volume (54) but not with gray matter and white matter volumes (54, 59). Similarly, higher VAT was linked to smaller temporal lobe and the volume of several other sub-compartments of the brain (76). Six studies (52, 54, 59, 62, 77, 78) have assessed the associations between VAT and White Matter Hyperintensities (WMH) which has been recognized as a risk factor for cognitive impairment and dementia (86). Among them, two have found a significant positive association, namely, associations of high VAT with greater volumes of WMH (62, 77). Similar associations were observed for higher VAT and greater vascular brain injury (52), or lower brain connectivity (fractional anisotropy [FA]), a measure of white matter integrity (74). Higher VAT was associated with higher damage in a brain network implicated in cognitive decline (53), suggesting that VAT is associated with accelerated brain aging (53). In contrast to this relatively broad evidence linking VAT with lower brain volumes and pathologies, two studies found associations of higher VAT with thicker cortex in the posterior cingulate gyrus (75), and with greater left cuneus volume (76).

**Table 4:** Brain changes and regional adiposity in studies included in the systematic review.

Brain changes	Brain parts	Studies	Regional fat depots	Association with regional fat depot	Difference
Volumetrics	Total brain volume	Isaac et al. (54)	VAT	−	NS
			SAT	+	NS
		Debette et al. (78)	SAT	−	*p* < 0.001
			VAT	−	*P* < 0.001
		Weinstein et al. (80)	NAFLD	−	*P* < 0.001
		VanWagner et al. (79)	LA	−	*p* < 0.05
	Frontal	Lee et al. (76)	VAT	+	NS
	Parietal	Lee et al. (76)	VAT	+	NS
	Temporal	Lee et al. (76)	VAT	−	*p* < 0.05
		Beller et al. (41)	VAT	+	NS
			Hepatic	−	NS
			Pancreatic	−	NS
	Occipital	Lee et al. (76)	VAT	+	NS
	Subcortical	Lee et al. (76)	VAT	−	NS
	Cerebellum	Lee et al. (76)	VAT	−	NS
	Gray Matter	Lee et al. (76)	VAT	−	NS
		Hsu et al. (59)	VAT	−	NS
			SAT	+	NS
			PAT	+	NS
		Raschpichler et al. (73)	VAT	−	*P* < 0.05
	White matter	Lee et al. (76)	VAT	+	NS
		Hsu et al. (59)	PAT	+	NS
			VAT	+	NS
			SAT	−	NS
	CSF	Lee et al. (76)	VAT	+	NS
	Hippocampal	Isaac et al. (54)	VAT	−	*P* < 0.05
			SAT	−	NS
		Hsu et al. (59)	PAT	−	NS
			VAT	−	*P* < 0.05
			SAT	−	NS
		Hsu et al. (59)	PAT	−	*P* < 0.05
			VAT	−	NS
			SAT	−	NS
		Weinstein et al. (80)	NAFLD	+	NS
		Beller et al. (41)	VAT	−	NS
			Hepatic	−	*P* < 0.05
			Pancreatic	−	NS
	Ventricular	Isaac et al. (54)	VAT	+	*P* < 0.05
			SAT	+	*P* < 0.05
	Cortical thickness	Isaac et al. (54)	VAT	−	*p* < 0.01
		Kaur et al. (75)	VAT	+	*P* < 0.05
	Temporal horn volume	Debette et al. (78)	SAT	+	NS
			VAT	+	NS
	Cingulate gyri	Beller et al. (41)	VAT	−	NS
			Hepatic	−	*P* < 0.05
			Pancreatic	−	NS
Wmhs	HWMHS	Anand et al. (52)	VAT	+	NS
		Isaac et al. (54)	VAT	+	NS
			SAT	−	NS
		Hsu et al. (59)	PAT	−	NS
			VAT	+	NS
			SAT	−	NS
		Pasha et al. (62)	VAT	+	*P* < 0.05
		Kim et al. (77)	VAT	+	*P* < 0.01
			SAT	+	NS
		Debette et al. (78)	SAT	−	NS
			VAT	−	NS
		Weinstein et al. (80)	NAFLD	−	NS
		Jang et al. (82)	NAFLD	+	*P* < 0.01
Brain infarcts	Vascular brain injury	Anand et al. (52)	VAT	+	*P* < 0.05
	Silent brain infarctions	Anand et al. (52)	VAT	+	NS
	Brain network covariance	Zsido et al. (53)	VAT	−	*P* < 0.01
	Fractional anisotropy	Cardenas et al. (74)	VAT	−	*P* < 0.05
	Lacunar infarct	Anand et al. (52)	VAT	+	NS
		Kim et al. (77)	VAT	+	*P* < 0.05
			SAT	+	NS
	Lacunes	Jang et al. (82)	NAFLD	+	NS
	Brain infarcts (BI)	Debette et al. (78)	SAT	−	NS
			VAT	−	NS
		Weinstein et al. (80)	NAFLD	+	NS
	Microbleeds	Jang et al. (82)	NAFLD	+	NS
Functional changes	Eigenvector centrality	Raschpichler et al. (73)	VAT	−	*P* < 0.01
	Cerebral blood flow	VanWagner et al. (79)	LA	−	*P* < 0.05
Ad-neuropathology	Tau pathology	Weinstein et al. (81)	NAFLD	+	NS
	Amyloid burden	Kim et al. (55)	VAT	+	*P* < 0.05
		Weinstein et al. (81)	NAFLD	+	NS

The table is ordered by brain changes. Significant findings are emphasized in bold.

### Visceral adiposity and AD-related pathology

The relationship of VAT with dementia pathology has been examined in two studies (55, 58). Individuals with higher VAT metabolism, a marker of higher VAT dysfunction assessed through PET-CT, compared with individuals with low VAT metabolism, exhibited significantly higher cerebral Aβ burden (55). Those findings suggest that VAT dysfunction could contribute to AD development. In a second study, using brain ^18^F-fluorodeoxyglucose (FDG) PET as a neurodegenerative biomarker for AD, computing the PALZ score a global index of AD-related hypometabolism, no significant correlation between VAT and the risk of AD pathology was found (58).

### Fatty liver and cognition

In the present study, we have identified seven studies examining the relationships of cognition and fatty liver or NAFLD (60, 67–72). In the past literature, we have identified three previous systematic review and meta-analyses (87–89) of observational studies, providing a comprehensive evaluation of the relationship between NAFLD and the risk of dementia or cognitive impairment. However, the studies included in our review include only the ones that have assessed liver fat and NAFLD with imaging technique. In most of the studies, higher severity of NAFLD was associated with increased risk of cognitive impairment (60, 67–71); in some cases, cardiovascular disease attenuated this relationship (60). Strong associations between NAFLD and worsening of cognition in patients meeting the criteria for vascular dementia was found (72). The impact of regional fat depots on cognitive functions can be found in Table 3.

### Fatty liver and brain changes

Five studies have reported the association between structural brain changes and fatty liver (41, 67, 79, 80, 82). Among them only one used a noninvasive functional measure, pseudo-Continuous Arterial Spin Labeling (pCASL) measuring cerebral blood flow (CBF) (79). The results are reported in Table 4. Hepatic fat depots were significantly associated with smaller total cerebral brain volume (79) as well as smaller cingulate gyri and hippocampal volumes (41). NAFLD was associated with a smaller total cerebral volume even after adjustment for VAT, pointing to a relationship between NAFLD and brain aging (80). In this study, no significant associations were observed between NAFLD and hippocampal and WMH volumes, nor with covert brain infarcts (small ischemic cerebral lesions here assessed by abnormal signal intensity) (80). Conversely, NAFLD was significantly associated with the presence of WMHs, even after adjusting for cardiometabolic risk factors (82) in another study. In NAFLD patients with lower cognitive score, the volumes of brain gray and white matter were significantly reduced compared to NAFLD patients with higher cognitive score (67). However, no comparison between the NAFLD patients and the control group without NAFLD volumes was done (67). Finally, higher liver fat shown by lower liver attenuation on abdominal CT was associated with decreased total-CBF and gray matter-CBF and this association remained after adjustment for cardiovascular risk factors (79).

### Fatty liver, AD-related pathology, and dementia

One study assessed NAFLD and AD-related neuropathology via PET-CT (81). Prevalent NAFLD was not associated with Aβ or tau PET, the main two pathologies characterizing AD (81). We found only one study investigating associations of NAFLD and dementia risk (66). Moderate-to-severe NAFLD was found to be associated with dementia and AD risk, especially with vascular dementia (66). Moreover, participants with vascular dementia and NAFLD had worse neuropsychological outcomes than participants without NAFLD (66).

### Other fat depots

Higher epicardial adipose tissue (EAT) has been associated with poorer cognitive functioning in two studies (64, 65). Pericardial adipose tissue (PAT) has been associated with lower hippocampal white matter but not hippocampal gray matter (59). One study examined associations of pancreatic fat with regional brain volume. Albeit in all analyses higher pancreatic fat was associated with lower hippocampal, cingulate gyri and temporal lobe volumes, none of these associations reached statistical significance (41). To our knowledge, no studies examining relationships of kidney fat depots with cognition or brain changes have been done.

### Factors potentially linking fat depots, brain changes and cognition

A few studies have explored inflammatory markers as possible factors linking fat depots, brain changes and cognition. Cannavale et al. (63), hypothesized that inflammatory markers would mediate the negative effect of VAT on selective attention. Indeed, plasma C-reactive protein (CRP) and Interleukin-6 (IL-6) concentrations mediated the relationship between higher VAT and lower attentional inhibitory control, suggesting that systemic inflammation could play a role in the deleterious effects of VAT on cognition (63). Higher levels of SAT and VAT were associated with worsening cognitive function in men even after controlling for metabolic disorders, adipocytokines (adiponectin, IL-6, tumor necrosis factor α [TNF-α], and plasminogen activator inhibitor-1[PAI-1]), and sex hormone levels (estradiol and testosterone) (56). Conversely, there was no association between adiposity and cognitive change in women (56). However, in another study where higher VAT was associated with lower cognitive functioning, estradiol level attenuated the negative consequences of VAT on cognition in women (53). Similar results were found for moderate-to- severe NAFLD, which was associated with increased serum levels of multiple cytokines, i.e., higher IL-6 concentrations partially mediated the association of moderate-to-severe NAFLD with vascular dementia (66).

Higher VAT and hepatic fat remained significantly associated with WMH (62, 82), decreased total-CBF and GM-CBF (79) and smaller brain volumes (41, 80) after adjustment for cardiovascular risk factors. Similarly, VAT was found to be significantly associated with reduced cognitive scores, after adjustment for cardiovascular risk factors, and for MRI-detected vascular brain injury (52). In several studies (60, 68, 69, 71) the association between NAFLD and cognitive impairment varied across the cognitive tests when adjusting for cardiovascular risk factors and diseases. Indeed, the NAFLD-cognitive function association was either attenuated (60, 71) or disappeared (68, 69, 71) when adjusting for these factors.

## Discussion

### Regional adiposity is associated with brain changes and higher risk of cognitive decline

Based on the current available literature, the findings indicate that different regional fat depots are likely associated with increased risk of cognitive impairment and dementia (36, 41, 52–82). Specifically, VAT (52, 54, 57, 61, 63), EAT (64, 65) and liver fat through NAFLD (60, 67–71, 79) were associated with cognitive impairment. Moreover, regional fat was linked to different brain changes (41, 52–63, 73–78), with a relatively consistent association of different fat depots with cortical volume (41, 54, 59, 73, 76, 78–80), and with white matter disease (62, 74, 77). Both lower cortical volume and white matter disease have been linked to cognitive decline, AD and dementia (90, 91). Interestingly, higher VAT, but not the other regional fat depots, was associated with amyloid β, a core neuropathological feature of AD (55, 81). This could be explained by the excessive secretion of leptin by high VAT, which in turn could inhibit the transport of Amyloid-β precursor protein and promote the fabrication of amyloid β (92). Finally, one of the studies has shown that greater severity of NAFLD was associated with higher risk of dementia (66), while among women higher SAT and thigh fat with lower likelihood of dementia (36). Overall, results from this systematic review suggest that different regional fat depots may lead to different neurobiological alterations and ultimately to different cognitive-related outcomes and dementia. Exploring potential mechanisms underlying the inter-relationships of regional adiposity- brain changes - cognition could lead to targeted and personalized treatments for cognitive-related outcomes.

### Metabolic syndrome and insulin resistance may link regional adiposity, brain changes, and cognition

The findings of associations of different regional fat depots with lower cognitive scores are concordant with previous research indicating that adiposity (assessed by BMI) is associated with cognitive impairment and risk of dementia (93). Central adiposity is a core feature of the metabolic syndrome (94) and has been associated with cognitive decline, dementia and neuropathology (94, 95), especially in old age (96). Yet, the metabolic syndrome has been associated with NAFLD and pancreatic fat independently of central obesity and insulin resistance (34, 97). Also, accumulation of VAT was found to be the best predictor for metabolic syndrome in women while it was a poor predictor for men compared to SAT (98). One possibility is that the impaired vascular function resulting from the different conditions of the metabolic syndrome could lead to brain changes that could then lead to cognitive impairment (95).

Another core feature of the metabolic syndrome is insulin resistance which also has been linked to cognitive decline and dementia (99). VAT rather than SAT is more strongly associated with insulin resistance (100) and NAFLD has also been closely linked to insulin resistance (101), showing that different fat compartments may be associated with differential metabolic risk. Although both VAT and fatty liver have been shown to be related to impaired cognition and both are determinants of insulin resistance, their impact is different due to the different roles played by adipokines and hepatokines, respectively (101). Those results imply the importance of assessing regional adiposity rather than central adiposity to understand the specific contribution of excess adiposity to cognition. Therefore, further studies should be done on regional fat depots to better understand the mechanism underlying the association between adiposity, metabolic syndrome, cognition, and brain changes. Investigation of regional fat in metabolically healthy obese population may shed light into these discrepancies (27, 28).

### Regional adiposity affects brain volumes and vasculature via systemic inflammation

In the present review, we have gathered data showing that regional fat depots are associated with deleterious brain changes. Regional fat depots including VAT, SAT and fatty liver were associated with smaller cerebral volumes (78–80). Fatty liver and VAT but not SAT were significantly associated with smaller hippocampal volume (41, 54, 59) which is one of the first regions affected by AD (90). Those results are concordant with recent evidence from our group showing associations of higher BMI with thinning of the middle temporal gyrus (102). Overall, the present review indicates that different fat depots can affect different parts of the brain suggesting a potential role of different regional fat depots in brain atrophy and pathology, targeting those fats could then prevent deleterious impact on the brain.

Brain small vessel disease which includes higher WMHs, and lacunar infarcts may lead to cognitive impairment and dementia (91). In the present review, different regional fat depots have also been associated with higher WMHs (62, 77, 82). In addition to its associations with cognitive impairment and AD (86), WMH is prevalent in individuals with insulin resistance, metabolic syndrome and T2D (103), all conditions accompanied by high adiposity (103). Furthermore, adiposity is associated with chronic low-grade systemic inflammation, which increases proinflammatory cytokine secretion (104). Pro-inflammatory cytokines have been linked to greater volumes of WMHs (104) which in turn are linked to cognitive impairment. As suggested by one of the studies reviewed, mechanisms by which VAT exerts a negative influence on cognitive function includes systemic inflammation (63). Therefore, disentangling factors secreted by different fat depots affecting systemic inflammation may shed light into their role in cognitive decline and dementia.

Indeed, different fat depots release different secreted factors, some of which cross readily the blood brain barrier (BBB) and may cause damage, ultimately leading to cognitive decline (56, 63, 66). For example, pro-inflammatory factors such as leptin, IL-6, TNF-α (105, 106) which are secreted by adipocytes can cross the BBB and lead to neuroinflammation, which plays a role in cognitive impairment and AD (107). Conversely, anti-inflammatory adipocytokines such as Adiponectin (108), Interleukin 10 (IL-10) (109, 110), and Apelin (111), are associated with less adiposity and are related to cognition and AD. Neuroinflammation likely causes synaptic remodeling and neurodegeneration resulting in disruption of cognitive functioning possibly resulting from damage brain regions subserving cognition such as the hippocampus (112). Targeting these factors could be an efficacious way to prevent or delay later cognitive decline and AD.

### Associations of adipose-secreted factors with neuropathology and impaired cognitive functioning

**Figure 2:**
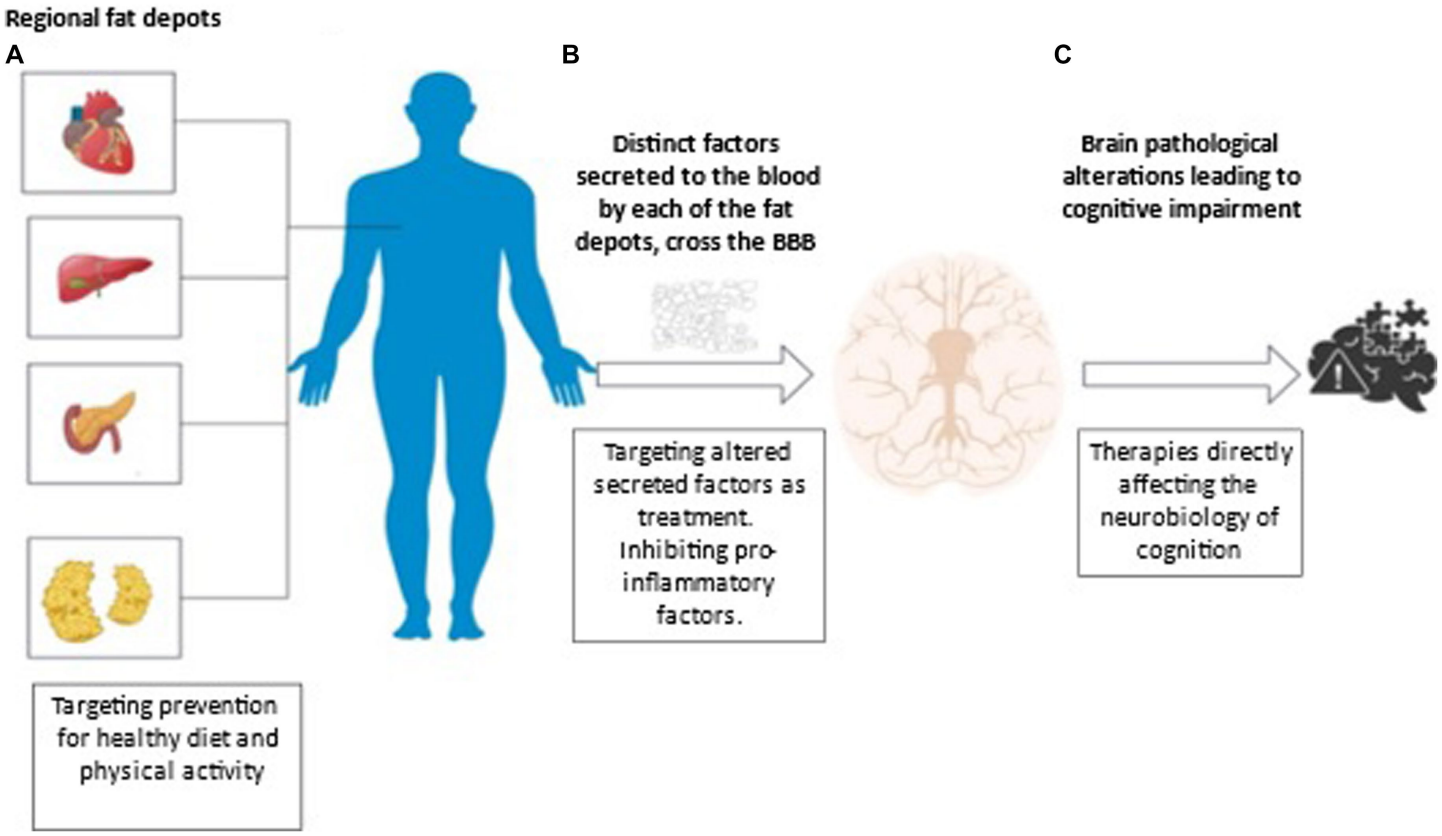
Potential mechanisms underlying the adiposity- brain changes - cognition inter-relationships and therapeutic modalities. **(A)** Adiposity is commonly used to describe excess body fat or obesity. Different fat depots can be found in the body independently of central obesity. Subcutaneous adiposity refers to the accumulation of fat underneath the skin, in the adipose tissue layer. Visceral adiposity refers to the accumulation of fat in the abdominal cavity, specifically around the organs such as the liver, pancreas, and intestines. Ectopic fat refers to the accumulation of fat in areas where it is not normally found, such as the liver, muscle, and pancreas. Both types of fat are associated with increased risk of metabolic disorders such as insulin resistance, type 2 diabetes, and cardiovascular disease. Understanding the association of adiposity, brain changes and cognitive abilities would provide key insights into the mechanisms by which adiposity impacts cognitive health and to possible treatments. Lifestyle interventions, such as specific diets and physical activity may diminish accumulation of fat depots. **(B)** Adiposity is associated with chronic low-grade systemic inflammation. Different fat depots can release different proinflammatory cytokines, hormones and enzymes some of which cross readily the blood brain barrier (BBB) and may cause damage ultimately leading to cognitive decline. For example, pro-inflammatory factors such as leptin, secreted by adipocytes, can cross the BBB and lead to neuroinflammation, which plays a major role in cognitive impairment and AD. Conversely, anti-inflammatory adipocytokines such as adiponectin, are associated with less adiposity and are related to better cognitive functioning and lower AD risk. Treatment targeting enhancing protective factors released from adipocytes and diminishing inflammatory factors secreted from fats could be an efficacious way to prevent brain damage and ultimately support healthy cognition. **(C)** Initial evidence suggests that different fat depots affect a plethora of brain pathologies including cortical volume, the cerebral vasculature, primarily white matter hyperintensities, and neuroinflammation. These pathologies contribute to cognitive impairment and as treatments addressing the neurobiology of cognition evolve, cognitive health may be maintained in spite of accumulation of fat deposits and their respective secreted factors.

Other factors, such as proteins are secreted from different fat depots, and may explain the role of peripheral fats in the brain. For example, Amylin, a hormone synthesized and co-secreted with insulin by pancreatic β-cells, is elevated in obesity and may share similar pathophysiology with Amyloid-β, characteristic of AD neuropathology (113). Also, Glucagon-like peptide-1 (GLP-1), a gut released hormone, which can protect pancreatic β-cells from apoptosis and induce insulin secretion, is attracting attention as a possible link between metabolic syndrome and brain impairment (114, 115). Additional factors, secreted by hepatocytes, the most common cells in the liver, are found to be related to cognition such as plasminogen activator inhibitor 1 (PAI-1) (116), and fetuin (117, 118). Indeed, in the presence of elevated fatty acids (119), circulating fetuin-A can induce insulin resistance (119) and inflammatory signaling (120) which may cause damage to the brain leading to cognitive impairment (117, 118). Therefore, identifying novel factors altered due to excess fat in different abdominal regions and associated with cerebrovascular pathology, neuropathology, and impaired cognitive functioning is crucial for developing fat-specific interventions. Potential mechanisms underlying the inter-relationships of adiposity- brain changes – cognition and therapeutic modalities, is presented in Figure 2.

### Sex differences in regional adiposity, brain changes, and cognition

Finally, sex differences should be taken into consideration as they may also contribute to the fat-brain-cognition axis. Women have overall more fat mass than men. Specifically, women have more SAT, which explains the “pear shape” (38), while they are characterized by lower VAT compared to men (121). Those differences in regional fat depots may lead to different consequences on cognition. Higher levels of VAT were associated with worsening cognitive function in men after adjustment for metabolic disorders, adipocytokines, and sex hormone levels (56). Conversely, there was no association between adiposity and cognitive changes in women (56). Furthermore, while VAT exacerbates the association between aging and poorer brain network covariance in both men and women, estradiol reduces the negative association in women (53). These findings highlight the need to account for sex differences in the investigation of relationships of regional adiposity with brain and cognition.

### Limitations

This review provides evidence for the association of different regional fat depots, cognition, and brain changes. However, this study had several limitations. First, the initial intention of our group was to conduct a meta-analysis on regional adiposity and cognition. But, due to the limited number of studies and the variability in methodologies the meta-analysis could not be conclusive as it carried high heterogeneity. The studies have different designs, sample sizes, and cognitive tests as well as different ways of assessing regional fat depots to quantify adiposity, adding complexity to the interpretation of results. Indeed, quantification of the degree of adipose tissues is different in each of the studies, as some quantify by fat volume and others by surface or percentage of fat in the different regions, making it difficult to directly compare the studies. Further prospective studies are needed to establish the relationship between regional fat depots with brain changes and cognition with similar methodologies. Only three studies in this review had longitudinal cognitive decline (56, 60, 68). Considering that the duration of exposure to adiposity may affect the onset and the severity of cognitive impairment, the lack of longitudinal data for regional adiposity is a significant limitation in the field. All studies were observational studies and not clinical trials therefore no causation can be inferred. In some studies, there were no associations between regional fat and cognition. However, many of these studies included relatively young individuals (e.g., 54.11 years, age at baseline for longitudinal studies). In such young ages the range of cognitive functioning is relatively narrow, possibly contributing to the lack of associations. It is important to note that the literature on fat and cognition may suffer from selection bias since older adults with cognitive impairment are less likely to participate in research. Finally, this review focuses specifically on body fat composition, rather than on general body composition, and does not discuss muscle mass and function which are strongly associated with cognitive decline and dementia risk (18–21).

## Conclusion

This review of 33 studies indicates that different regional fat depots may affect cognition and different regions of the brain. Regional fat depots, especially VAT and hepatic fat, have been associated with cognitive decline, cortical thinning and WMHs. Regional fat depots, rather than central obesity, may better explicate the association between adiposity and brain and may open horizons for new personalized fat-reducing treatments for prevention of cognitive decline.

## Author contributions

EB, SG, and MB conceived the presented idea and have made a substantial contribution to the concept and design of the manuscript. EB carried out the literature search from electronic databases, drafted the manuscript, and provided the tables. SG participated with EB to the full-text screening of the articles from the literature search, read and approved the manuscript. MB revised critically the manuscript and approved the version to be published. All authors contributed to the article and approved the submitted version.

## Funding

This work was funded by National Institutes of Health grants R01-AG-034087, AG-053446, and AG-051545 (to MB).

## Conflict of interest

The authors declare that the research was conducted in the absence of any commercial or financial relationships that could be construed as a potential conflict of interest.

## Publisher’s note

All claims expressed in this article are solely those of the authors and do not necessarily represent those of their affiliated organizations, or those of the publisher, the editors and the reviewers. Any product that may be evaluated in this article, or claim that may be made by its manufacturer, is not guaranteed or endorsed by the publisher.
